# SARS-Cov2 S Protein Features Potential Estrogen Binding Site

**DOI:** 10.17113/ftb.59.01.21.6820

**Published:** 2021-03

**Authors:** Ante Tomasović, Damir Stanzer, Ivan Krešimir Svetec, Marina Svetec Miklenić

**Affiliations:** Faculty of Food Technology and Biotechnology, University of Zagreb, Pierottijeva 6, 10000 Zagreb, Croatia

**Keywords:** SARS-CoV2, coronavirus, S protein, estrogen, estradiol, estrogen binding site

## Abstract

**Research background:**

During the current SARS-CoV2 pandemic, as well as earlier SARS and MERS epidemics, it has been observed that COVID19-positive women on average tend to have milder symptoms and lower fatality rates than men. There is a number of differences between the sexes known to contribute to different immune responses and severity of the disease, one being the effect of estrogen *via* estrogen receptor signalling. We wondered if estrogen might also affect the SARS-CoV2 more directly, perhaps by binding to the surface glycoprotein (S protein), thus possibly reducing its infectivity.

**Experimental approach:**

To assess whether there is a possibility for estrogen binding on the SARS-CoV2 S protein, we used BLAST and HHpred to compare protein sequences of S protein and human estrogen receptor β, while 3D structures of a potential estrogen binding site and an active site of estrogen receptor β were visualized and compared using PyMOL.

**Results and conclusions:**

By comparing the sequence of SARS-CoV2 S protein with the human estrogen receptor β, we identified a potential estrogen binding site on S protein and further determined that it also shares notable similarities with the active site of ER β when observed in 3D structure of their respective proteins. As a control, SARS-CoV2 S protein was compared with the human androgen receptor, and no such similarities were found. The potential estrogen binding site is part of coronavirus S2 superfamily domain, which is involved in host-virus membrane fusion during infection and appears to be conserved throughout the *Coronaviridae* family.

**Novelty and scientific contribution:**

This preliminary communication shows that SARS-CoV2 S protein features a potential estrogen binding site. Hopefully, this will prompt a more comprehensive study on the possibilities of estrogen binding on the S protein and the effect this might confer on the virus infectivity.

## INTRODUCTION

The COVID-19 pandemic that has resulted in high numbers of critically ill patients and deaths is caused by novel coronavirus SARS-CoV2. Studies on the infection and clinical management reported sex differences in severity and outcome of the COVID-19 disease (*1*-*7*). It is known that males are more susceptible to influenza and common cold than females of the same age (*8*-*10*). Two other coronaviruses that also cause severe respiratory illness as SARS-CoV-2 does, severe acute respiratory syndrome coronavirus (SARS-CoV) and the Middle East respiratory syndrome coronavirus (MERS-CoV) with a nucleotide identity to SARS-CoV-2 of 79 and 50% respectively (*11*), also tend to be more severe and fatal in men than in women (*12*-*15*).

Besides lifestyle factors, the sex-specific difference in coronavirus susceptibility and disease outcomes is caused by sex differences in the immune response, both innate and adaptive (*16*). One of the explanations for this sex difference in COVID-19 susceptibility is chromosomal: X chromosome contains more immune-related genes than the Y chromosome, and activation of these genes leads to stronger female immune response to viral infection (*7*, *17*, *18*). Another explanation is hormonal: hormones, such as estrogen, which females produce in larger quantities than males, help to defend against coronaviruses like MERS-CoV, SARS-CoV and SARS-CoV-2. There are various possible explanations of estrogen protective action mechanism (*19*-*24*). Among the effects of estrogen in the immune defence, there is an influence on adaptive immunity fighters, T- and B-cells, by impairing negative selection of high affinity auto-reactive B cells, modulating B cell function and leading to Th2 response (*25*), and induction of T cell homing by enhancing the expression of CCR5, a homing marker (*26*). An experiment performed with mice infected with mouse-adapted SARS-CoV MA15 (*27*) showed that the male mice were more susceptible to the SARS infection than females, and that the female mice that had their estrogen-producing ovaries removed or were treated with an estrogen-receptor blocker had higher fatality rates than those with normal estrogen function. The sex-specific differences were independent of T and B cell responses. Therefore, these results suggest that estrogen receptor (ER) signalling in females suppressed the accumulation and function of inflammatory monocyte macrophages in the lungs and/or directly suppressed SARS-CoV replication *via* effects on cellular metabolism.

Besides acting on ER signalling as a mechanism of conferring strong positive effect on disease outcomes and fatality rate (*25*), we considered the idea that the estrogen might act directly on the virus. We hypothesized that the estrogen molecule might bind on the virus surface and thus reduce its infectivity. Since spike (S) protein is the major surface protein the SARS-CoV2 employs to bind to the human receptor and initiate fusion with the host cell (*26*-*29*), we wondered if there was any theoretical possibility that estrogen could bind to this protein. If this were indeed the case, then estrogen and estrogen-like molecules might have a therapeutic effect. To get some insight into these questions, we first performed protein BLAST to determine if there was any similarity between SARS-CoV2 S protein and the human ER β sequences. Although these two proteins originate from different species and differ greatly in their overall size, a sequence similarity was found in regions which correspond to the estrogen binding site of the human ER β. We further explored the spatial structure of potential estrogen binding site on S protein using PyMol software and again found substantial similarities with the 3D structure of binding site on the human ER β.

## MATERIALS AND METHODS

### Protein sequences and 3D structures

The following protein sequences were downloaded from NCBI (*30*): human ER β sequence (UniProtKB/Swiss-Prot: Q92731.2), human androgen (dihydrotestosterone) receptor (UniProtKB/Swiss-Prot: P10275.3), SARS-CoV2 surface glycoprotein (NCBI reference sequence: YP_009724390.1) and SARS CoV Urbani spike protein (GenBank: AYV99817.1). The following 3D structures were downloaded from RCSB Protein Data Bank (*31*): crystal structure of ER β bound to estradiol (PDB ID: 5TOA (*32*)), crystal structure of human androgen receptor ligand-binding domain in complex with dihydrotestosterone (PDB ID: 2AMA (*33*)) and prefusion 2019-nCoV spike glycoprotein with a single receptor-binding domain up (PDB ID: 6VSB (*29*)).

### Bioinformatic tools

Protein sequence alignment and analysis were performed using protein BLAST (*34*) and the HHpred server for remote protein homology detection and structure prediction (https://toolkit.tuebingen.mpg.de/tools/hhpred (*35*)). In all cases, standard algorithm parameters were used (*36*, *37*). For 3D protein structure, visualization the PyMOL software was used (*38*).

## RESULTS AND DISCUSSION

To assess whether there could be any possibilities for the S protein of SARS-CoV2 to contain a site that might bind estrogen, we used protein BLAST to compare its sequence with human ER β. We found three regions of similarity. The two more significant were: a region of 19 amino acids starting from amino acid 319 of the ER β and amino acid 817 of S protein with 73% positives without gaps (e-value 2.5) and a region of 14 amino acids starting from amino acid 385 of the ER β and amino acid 236 of S protein with 64% positives without gaps (e-value 3.2). Although the e-values were relatively high, we found it interesting that both of these two regions are annotated in NCBI as part of the ligand-binding domain of the estrogen receptor.

**Fig. 1:**
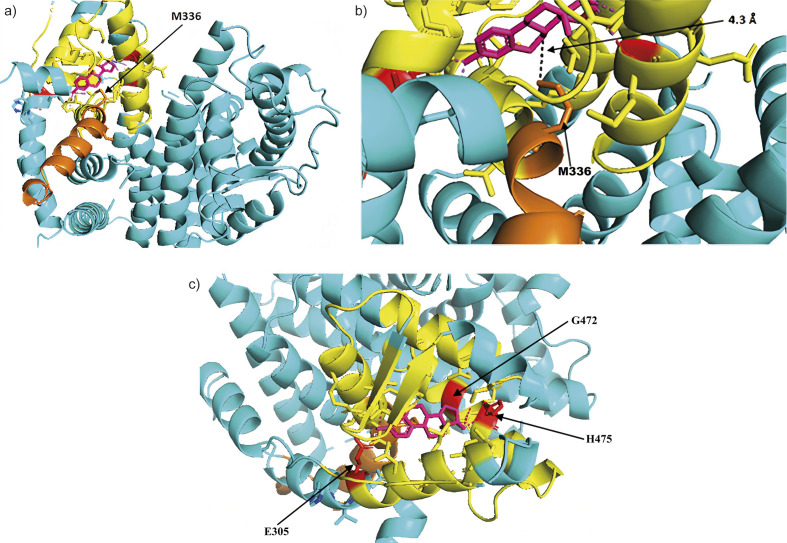
The 3D structure of: a) human estrogen receptor β in the vicinity of 19 amino acid sequence alignment with the S protein spanning residues from F319 do E337 (orange) with estradiol (magenta) bound to the active site, b) the alignment ends with M336 protruding into the active site and being less than 5 Å from the estradiol molecule, and c) the active site seen from different perspective offers better view of other surrounding residues likely involved in binding (yellow) and the three residues (E305, G472 and H475, marked red) which can form polar bonds with estradiol

Furthermore, we visualized the 3D structure of the SARS-CoV2 S protein, using PyMOL software (*38*), and compared it to the 3D structure of ER β to check whether the regions of S protein carrying either the 19 or 14 amino acid alignments found by BLAST bear any spatial resemblance to the estrogen binding site of ER β and thus could constitute a potential estrogen binding site on the S protein. According to the 3D structure analysis, the 19 amino acid region (319F to 337E) appears to play an important role in the ligand-binding domain on the ER β (Fig. 1a) since it protrudes into the active site with the methionine 336M as the closest amino acid to the estradiol (a major form of estrogen in women of reproductive age) when bound. The residue 336M is less than 5 Å apart from the estradiol molecule (Fig. 1b). Therefore, it is reasonable to assume that the terminal part of the 19 amino acid alignment constitutes a part of an active site on ER β. Residues involved in ligand-binding on ER β are glutamate 305E situated on the first alpha helix and the glycine 472G and histidine 475H situated on the second alpha helix, which form three polar bonds with estradiol (Fig. 1c).

**Fig. 2:**
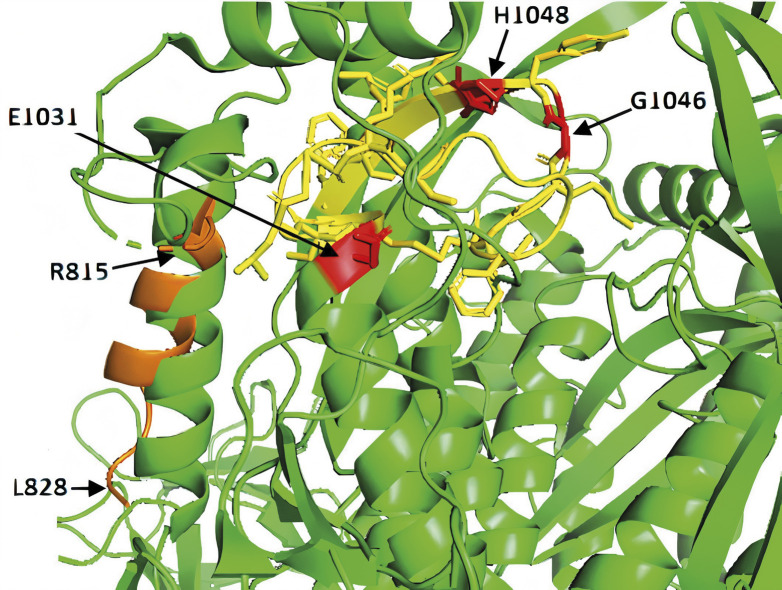
The 3D structure of S protein in the vicinity of potential estrogen binding site. The 19 amino acid sequence alignment with the region on ER β is shown in orange. Residues R815 and L828 are sites of protein chain breakage. Amino acids possibly involved in polar binding of estrogen in the potential active sites (E1031, G1046 and H1048) are marked red while other residues surrounding the potential binding site are shown in yellow

In the SARS-CoV2 S protein, the 19 amino acid alignment begins with phenylalanine 817F. In the corresponding 3D structure obtained by electron micrograph (*29*) residues 817F through 828L can be found. Unfortunately, the structure contains a brakeage of protein chain between the residues 811 and 815 as well as 828 and 853 resulting in separation of this part of the molecule. Moreover, the protein chain breakage visible in the 3D structure of SARS-CoV2 S protein is present at the approximately same site in the SARS-CoV Urbani 3D structure (the related coronavirus causing SARS pandemic in 2003), indicating that this particular site is sensitive to sample preparation and manipulation. However, the separated alpha helix is located near the breakage site, indicating that it has not moved considerably from its native position (Fig. 2). Instead of methionine 336M present in the analyzed 19 amino acid region on ER β, the corresponding region on S protein contains an analogue nonpolar residue isoleucine 834I scored as positive by BLAST. Although the exact position of 834I in the available model of S protein cannot be observed due to the protein chain breakage, it can be positioned in space based on the surrounding residues. We then tried to identify the amino acids that might be close in space to the 834I and could form polar bonds with estradiol molecule. Using BLAST, we identified a region of S protein (residues 1045 to 1052), which aligns with the region of ER β spanning residues 471 to 479 with 66% of them scored as positives, and which contains two key residues for estradiol binding on ER β – 472G and 475H. The main difference is that on the S protein the glycine and histidine are separated by one amino acid (Y), while two residues in between are present on the ER β (M and E). To find the potential analogue of the third amino acid responsible for estradiol binding on ER β (305E), we used the HHpred server (*35*). The region spanning residues 289 to 310 of ER β (containing 305E) was compared against the entire sequence of S protein. The analysis resulted in region spanning residues from 1013 to 1033 of S protein identified as similar with two gaps introduced to achieve the alignment of 305E with its potential analogue on the S protein – the glutamate at position 1031.

**Fig. 3:**
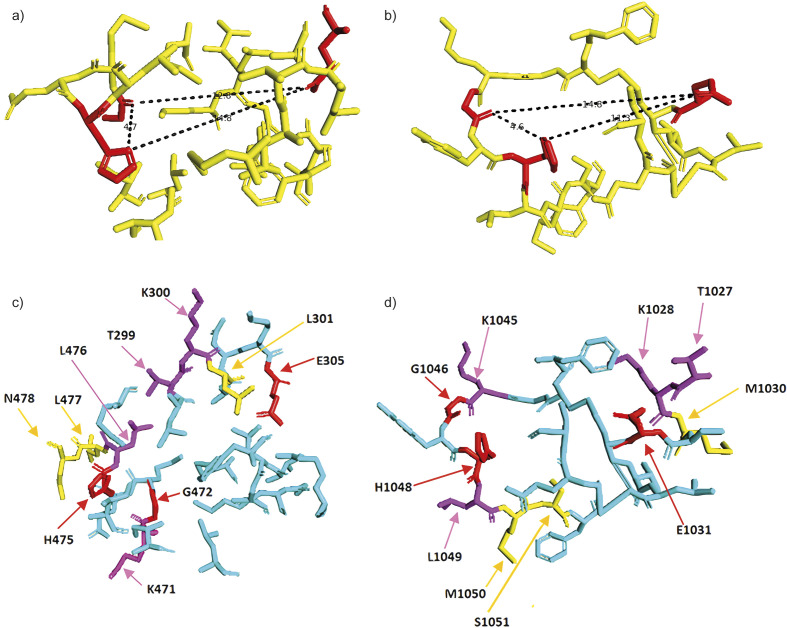
Comparison of estrogen binding site of ER β and potential estrogen binding site found on S protein of SARS-CoV2. Amino acids E, G and H capable of forming polar bonds with estrogen are depicted red: a) sides of the triangle between E, G and H residues are 4.7, 12.8 and 14.8 Å on ER β, and b) 4.6, 14.8 i 11.3 Å on S protein, c) identical (red and purple) and similar (yellow) residues surrounding the estrogen binding site of ER β compared with d) potential estrogen binding site on SARS-CoV2 S protein

Finally, using PyMol (*38*), we visualized the spatial position of three amino acids, which can form polar bonds with the ligand (1046G, 1048H and 1031E) on the 3D structure of S protein and compared this site to the active site for estradiol binding on ER β. Histidine residue 1048H on the S protein was rotated in space around alpha C atom to face towards the 1031E, which is likely to occur in the case of estradiol binding. The three key amino acids on the ER β responsible for polar binding of estradiol form a triangle with the length of its sides being 4.7, 12.8 and 14.8 Å (Fig. 3a). On the S protein the three analogous amino acids form a triangle with sides 4.6, 14.8 and 11.3 Å long (Fig. 3b). In other words, the dimensions of the space between the residues capable of forming polar bonds with estradiol in ER β and the analyzed site on S protein are curiously similar, with the main difference being that the longest side of the triangle in the active site of ER β is between histidine and glutamate and of the S protein between glycine and glutamate. Moreover, besides the three residues with the potential of forming polar bonds with estradiol, a number of other residues surrounding the potential binding site on S protein are identical or similar in their chemical properties to that present in the ER β active site (Figs. 3c and 3d).

As a control, sequences and 3D structures of S protein and human androgen receptor were compared using the same methodology as described above. Although testosterone and estrogen are highly similar molecules and the estrogen and testosterone receptors share regions of notable similarity in the ligand-binding domain, we did not find sequence alignments of S protein with the androgen receptor that include all three key residues for testosterone binding (R, T and N), nor could we identify a site in the 3D structure of S protein that would share significant resemblance to the testosterone binding site (data not shown).

Taken together, our analyses suggest that SARS-CoV2 S protein features a potential estrogen binding site. Understandably, whether or not the estradiol molecule actually can bind to the potential site - and if it does bind, how it affects the S protein and the infectivity of the virus can be established only experimentally. The strong positive effect of estrogen on disease outcome and fatality rate was experimentally shown in mice using mouse-adapted SARS-CoV MA15 and the effect was explained by estrogen receptor signalling (*27*). The entire S protein of mouse-adapted strain differs in only one amino acid from the SARS-CoV, the human virus causing the SARS 2003 epidemic (*27*). Thus, we wondered if SARS-CoV, which is closely related to SARS-CoV2, also features a potential estrogen binding site and whether it is conceivable that at least to an extent the positive effect of estrogen observed in these experiments might be due to the S protein binding estrogen. The BLAST analysis revealed that in the region 800–1273, which includes the potential estrogen binding site, the two S proteins are 93% identical (with 97% amino acids scored as positives without gaps). The 3D structure of SARS-CoV also presented the same conformation in the investigated region with the potential estrogen binding site present (data not shown).

**Fig. 4:**
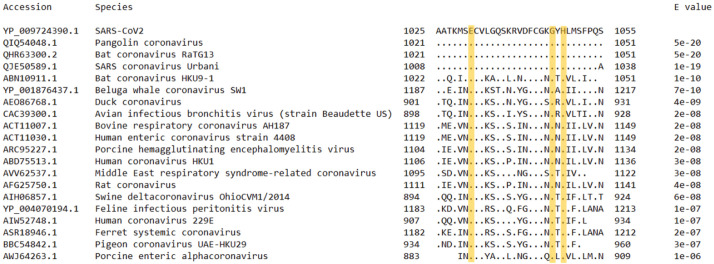
Results of BLAST analysis of SARS-CoV2 S protein region from 1025 to 1055 against S proteins of 20 randomly chosen species across the *Coronaviridae* family. Dots in the alignment represent identical amino acids. Yellow squares outline the key amino acids (E, G and H) identified as having potential for estradiol binding on SARS-CoV2 S protein

Moreover, the entire region of SARS-Cov2 S protein ranging from residue 662 to 1270 is a conserved S2 domain belonging to the coronavirus S2 superfamily. After transcription, the S protein is cleaved to S1 and S2 subunits. The S1 is responsible for binding to the host receptor, while the S2 subunit contains machinery for fusion with the host cell which consists of several distinct regions: the fusion peptide, heptad repeats 1 and 2 (HR1 and HR2) and transmembrane domain (*39*). The potential estrogen binding site identified in this work lies in the region carrying a linker between the two heptad repeats (*40*). A BLAST analysis of SARS-CoV2 S protein region 1025 to 1055 against S proteins of 20 randomly chosen species across the *Coronaviridae* family showed that this site is conserved, with the three amino acids (E, G and H) likely capable of forming polar bonds with estradiol together with some of the surrounding amino acids being present across the family (Fig. 4). Given the similarities of this region to the ER β, it is possible that an ancestral viral species at some point in time integrated a section of an ER from its host (*41*). However, it seems that this section might have taken on an important role in the coronavirus infectivity as a part S2 domain, perhaps being involved in host-virus membrane fusion by binding of other molecules featuring four rings, such as cholesterol. Thus, binding of estrogen might compete with the natural ligand for this site or cause conformational changes leading to reduced function of S2 domain and diminished infectivity.

Given the differences in COVID19 disease outcomes and fatality rate between men and women, the usage of estradiol to reduce severity of COVID19 infection is currently being tested in a phase II clinical trial in Stony Brook University Hospital, New York, NY, USA (https://clinicaltrials.gov/ct2/show/NCT04359329). The positive and suspected to be positive patients are given estradiol patches delivering 100 μg/day of estradiol for 7 days and rates of hospitalization, transfer to intensive care unit, intubation and death are being monitored during the 30-day time frame and compared to the non-treated control. The study recruits men over the age of 18 and women over the age of 55. Hopefully, although preliminary, the results presented in this work will inspire more researchers into investigating estrogen and estrogen-like molecules for treating COVID-19 since using different drugs, doses and methods of delivery could all affect the final result.

## CONCLUSIONS

In this work, we identified a conserved potential estrogen binding site on SARS-CoV2 surface glycoprotein (S protein). This result could offer additional explanation of the better disease outcomes for COVID19 positive women than men, as well as of the strong protective effect of estrogen in experimental animals. Most importantly, this result could prompt other researchers to experimentally prove whether or not the estrogen can indeed bind to the S protein and what effect would that confer on the infectivity of the virus. Hopefully, estrogen or estrogen-like molecule might be used to treat SARS-CoV2 infection or alleviate symptoms.
